# Supplementary material for: Infections of Venetoclax-Based Chemotherapy in Acute Myeloid Leukemia: Rationale for Proper Antimicrobial Prophylaxis
Source: Cancers (Basel). 2021 Dec 14;13(24):6285. doi: 10.3390/cancers13246285 (PMC8699304; doi:10.3390/cancers13246285)
Supplement: Supplementary file 1 [file cancers-13-06285-s001.zip › cancers-1478677-supplementary.pdf]

Supplementary materials: Infections of Venetoclax-Based Chemotherapy in Acute Myeloid Leukemia: Rationale for Proper Antimicrobial Prophylaxis

Raeseok Lee, Sung-Yeon Cho, Dong-Gun Lee, Hyeah Choi, Silvia Park, Byung-Sik Cho, Yoo-Jin Kim and Hee-Je Kim

Table S1. Characteristics of patients with invasive fungal infections by the time of diagnosis.

Variables	Early Onset IFIs=17 (Number, %)	Late Onset IFIs=5 (Number, %)	Total IFIs=22 (Number, %)	*p*-Value
Sex (male)	11 (64.7)	2 (40.0)	13 (59.1)	0.638
Age, years (median, IQR)	61 (48–67)	67 (66–77)	62 (49–71)	0.114
AML type at diagnosis				0.592
De novo/MRC	11 (64.7)	2 (40.0)	13 (59.1)	
Secondary	5 (29.4)	2 (40.0)	7 (31.8)	
Therapy-related	1 (5.9)	1 (20.0)	2 (9.1)	
AML status at initiation of VEN-based therapy				1.000
Newly diagnosed	5 (29.4)	1 (20.0)	6 (27.3)	
Refractory/relapsed	12 (70.6)	4 (80.0)	16 (72.7)	
AML risk group				0.877
Favorable	5 (29.4)	1 (20.0)	6 (27.3)	
Moderate	7 (41.2)	2 (40.0)	9 (40.9)	
Poor	5 (29.4)	2 (40.0)	7 (31.8)	
Combination agents				0.557
Decitabine	15 (88.2)	4 (80.0)	19 (86.4)	
Azacitidine	1 (5.9)	1 (20.0)	2 (9.1)	
Low-dose cytarabine	1 (5.9)	0 (0.0)	1 (4.5)	
Overall response				0.792
Response	8 (47.1)	3 (60.0)	11 (50.0)	
Non response	8 (47.1)	2 (40.0)	10 (45.5)	
Not available	1 (5.9)	0 (0.0)	1 (4.5)	
Total cycle of VEN-based therapy (median, range)	2.0 (1.0–3.0)	3.0 (3.0–4.0)	2.0 (2.0–3.0)	0.008
Type of antifungal agents at development of IFIs				1.000
Fluconazole	15 (88.2)	4 (80.0)	19 (86.4)	
Mold active antifungal agents	2 (11.8)	1 (20.0)	3 (13.6)	
Prolonged neutropenia	16 (94.1)	3 (60.0)	19 (86.4)	0.225
Profound neutropenia	15 (88.2)	3 (60.0)	18 (81.8)	0.436
Response at development of IFIs				0.572
Response	8 (47.1%)	1 (20.0)	9 (40.9)	
Non response	9 (52.9%)	4 (80.0)	13 (59.1)	
Overall mortality	12 (70.6%)	2 (40.0)	14 (63.6)	0.471

AML: acute myeloid leukemia; IFIs: invasive fungal infections; IQR: interquartile range; MRC: myelodysplasia-related changes; VEN: venetoclax.

Table S2. A, Microbiologic classification of bloodstream isolates and antimicrobial susceptibility patterns of the gram-positive organisms and B, Microbiologic classification of bloodstream isolates and antimicrobial susceptibility patterns of the gram-negative organisms.

**A. Pathogens (No. of Isolates)**	**No. of Isolates Susceptible to Antibacterial Agents/No. of Isolates Tested**																	
**Gram-Positive Bacteria**	**AMC**	**AMP**	**CTX**	**ERY**	**LZD**	**SYN**	**CIP**	**CLI**	**FA**	**GEN**	**NIT**	**OXA**	**PEN**	**RIF**	**TEC**	VAN	TET	TGC	TMP/SMX
Enterococcus faecalis (3)	1/3	1/3		0/3	3/3	0/3							0/3		3/3	3/3		3/3	
Enterococcus faecium (3)	0/3	0/3		0/3	3/3	3/3							0/3		0/3	0/3		3/3	
Staphylococcus aureus (1)				1/1	1/1		1/1	1/1	0/1	1/1	1/1	1/1	0/1	1/1	1/1	1/1	1/1	1/1	1/1
Staphylococcus epidermidis (2)				0/2	2/2		0/2	1/2	1/2	0/2	2/2	0/2	0/2	2/2	2/2	2/2	1/2	2/2	1/2
Streptococcus mitis/oralis (1)		1/1	1/1	1/1	1/1			1/1					1/1			1/1	1/1	1/1	
Corynebacterium (4)							0/4							2/4		4/4	1/4		
Micrococcus luteus (1)																			

**B. Pathogens (No. of Isolates)**	**No. of Isolates Susceptible to Antibacterial Agents/No. of Isolates Tested**																	
**Gram-Negative Bacteria**	**AML**	**GEN**	**AMC**	**AMP**	**ATM**	**CFZ**	**FEP**	**CTX**	**FOX**	**CAZ**	**CIP**	**ESBL**	**ETP**	**IPM**	**MEM**	**TZP**	TGC	TMP/SMX
Escherichia. coli (10)	10/10	7/10	7/10	2/10	8/10	3/10	10/10	8/10	10/10	9/10	2/10	2/10	9/10	10/10	10/10	9/10	10/10	5/10
Klebsiella pneumoniae (6)	6/6	4/6	4/6	0/6	5/6	3/6	5/6	3/6	6/6	5/6	4/6	3/6	6/6	6/6	6/6	5/6	6/6	6/6
Pseudomonas aeruginosa (3)	3/3	3/3			2/3		3/3			3/3	3/3			3/3	3/3	3/3		
Enterobacter cloacae (2)	2/2	2/2	0/2	0/2	2/2	0/2	2/2	2/2	0/2	2/2	2/2		2/2	2/2	2/2	2/2	2/2	2/2
Acinetonacter baumannii (1)	1/1	1/1	1/1			0/1	1/1	1/1		1/1	1/1			1/1	1/1	1/1	1/1	1/1
Fusobacterium nucleatum (1)																		

Leptotrichia trevisanii (1)	

AMC: amoxacillin-clavulanate; AMK: amikacin; AMP: ampicillin; ATM: aztreonam; CAZ: ceftazidime; CFZ: cefazolin; CIP: ciprofloxacin; CLI: clindamycin; CTX: cefotaxime; ERY: erythromycin; ESBL: Extended-Spectrum β-Lactamase; ETP: ertapenem; FA: fusidic acid; FEP: cefepime; FOX: cefoxitin; GEN: gentamicin; IPM: imipenem; LZD: linezolid; MEM: meropenem; NIT: nitrofurantoin; No: number; OXA: oxacillin; PEN: penicillin; RIF: rifampin; SYN: quinupristin and dalfopristin; TEC: teicoplanin; TET: tetracycline; TGC: tigecycline; TMP/SMX: trimethoprim/sulfamethoxazole; TZP: piperacillin-tazobactam; VAN: vancomycin.
